# Prevalence and predictors of Motoric Cognitive Risk syndrome in a community‐dwelling older Scottish population: A longitudinal observational study

**DOI:** 10.1002/gps.5824

**Published:** 2022-10-06

**Authors:** Donncha S. Mullin, Lucy E. Stirland, Miles Welstead, Tom C. Russ, Michelle Luciano, Graciela Muniz‐Terrera

**Affiliations:** ^1^ Alzheimer Scotland Dementia Research Centre University of Edinburgh Edinburgh UK; ^2^ Edinburgh Dementia Prevention Group University of Edinburgh Edinburgh UK; ^3^ Division of Psychiatry Centre for Clinical Brain Sciences University of Edinburgh Edinburgh UK; ^4^ NHS Lothian Royal Edinburgh Hospital Edinburgh UK; ^5^ Lothian Birth Cohorts Department of Psychology University of Edinburgh Edinburgh UK; ^6^ Department of Social Medicine Ohio University Athens Ohio USA; ^7^ BrainLat Universidad Adolfo Ibanez Penalolen Chile

**Keywords:** dementia, Frailty, Mild Cognitive Impairment, Motoric Cognitive Risk, slow gait, subjective cognitive complaint

## Abstract

**Objectives:**

Motoric Cognitive Risk (MCR) is a gait‐based predementia syndrome that is easy to measure and prognostic of dementia and falls. We aimed to examine the prevalence and risk factors for MCR, and assess its overlap with Mild Cognitive Impairment, Prefrailty, and Frailty, in a cohort of older Scottish adults without dementia.

**Methods:**

In this longitudinal prospective study, we classified 690 participants (mean [SD] age 76.3 [0.8] years; wave 3) of the Lothian Birth Cohort 1936 (LBC1936) into non‐MCR or MCR groups. We examined their baseline (age 69.5 [0.8] years; wave 1) risk factors for MCR at waves 3, 4, and 5 (6, 9, and 12 years later respectively).

**Results:**

MCR prevalence rate ranged from 5.3% to 5.7% across the three waves. The presence of MCR was associated with older baseline age (6 and 9 years later), lower occupational socioeconomic status (6 years later), and worse scores in a range of tests of executive function (6, 9 and 12 years later). Approximately 46% of the MCR group also had Mild Cognitive Impairment, and almost everyone in the MCR group had either Prefrailty or Frailty.

**Conclusions:**

The prevalence of MCR in this Scottish cohort is lower than the pooled global average, possibly reflecting the general good health of the LBC cohort. However, it is higher than the prevalence in two neighbouring countries' cohorts, which may reflect the younger average ages of those cohorts. Future LBC1936 research should assess the risk factors associated with MCR to validate previous findings and analyse novel predictive factors, particularly socioeconomic status.

## Key points

- Motoric Cognitive Risk (MCR) is a gait‐based predementia syndrome that is quick, inexpensive, and practical to assess and diagnose, and it can identify individuals at high risk of developing dementia.
- The prevalence of MCR in this older Scottish cohort ranged from 5.3% to 5.7% over three follow‐up waves.
- Factors associated with MCR in this cohort include age, socioeconomic status and tests of executive function.
- There is partial overlap between individuals with MCR and Mild Cognitive Impairment, but almost all individuals with MCR also had either Prefrailty or Frailty.

## 1 INTRODUCTION

Dementia is a leading cause of morbidity and mortality globally.^1^ Effective treatments for dementia remain elusive. There is a pressing need to identify adults at high risk for dementia. This would enable the implementation of risk‐modifying interventions based on lifestyle, organising future care needs, and assisting with cohort recruitment to trials. All of this could ultimately contribute to a reduction in the prevalence of dementia.^2^ The Motoric Cognitive Risk (MCR) syndrome is a high‐risk predementia state combining objective (measured) slow walking speed and subjective (self‐reported) cognitive complaint in the absence of significant functional impairment and dementia.^3^ Slow gait speed and subjective cognitive complaints are some of the earliest reported findings in the pre‐clinical stage of dementia, occurring approximately 10 years before dementia diagnosis.^4^ MCR is a better predictor of dementia than its individual components of slow gait or cognitive complaint alone.^3^, ^5^

MCR is quick, inexpensive, and practical to assess and diagnose. It does not require any expensive technology, specialised assessment, invasive investigations, or brain imaging scans. Thus, MCR could be useful in low‐ and middle‐income countries, where currently two‐thirds of the global population with dementia reside,^1^ while also having the potential to be an adjunct to memory services referrals in more economically developed countries.

MCR is a recently defined construct, first appearing in the literature in 2013.^3^ As such, and despite a growing body of literature on MCR, it is important to determine its prevalence and associated factors in diverse global populations. To date, prevalence rates range from 1.7%^6^ (Australia) to 27% (India).^7^ Generally, higher‐income countries have lower prevalence rates of MCR, although how the MCR criteria are operationalised across studies also affects rates.^8^ An increasing body of work supports the prognostic utility of MCR. A 2022 systematic review and meta‐analysis showed that, compared to individuals without MCR, individuals with MCR are at over twice the risk of developing dementia after 4.3 years of follow‐up.^9^ It also reported that the MCR group were 76% more likely to develop cognitive impairment and that MCR is prognostic of future falls and earlier mortality.^9^

This is the first study to derive MCR in a Scottish cohort and determine its prevalence. In doing so, it is the first study to report on slow gait cut‐scores in this population. We assess for risk factors which have previously been associated with MCR^8^, ^10^, ^11^ except for early life IQ, which has not been tested until now. Another novelty is that this study explores the overlap between MCR and the other high‐risk predementia states of Mild Cognitive Impairment (MCI), Prefrailty, and the Fried Frailty phenotype. Recent work found that a 41‐point cumulative deficit frailty index increased the risk of incident MCR.^12^ By evaluating the overlap of MCR with the Fried Frailty phenotype and the earlier state of Prefrailty, we hope to clarify the degree of cross‐over between these related states, highlighting areas of convergence and divergence.

This study aims to determine the prevalence of MCR syndrome, describe associated risk factors, and assess its overlap with MCI, Prefrailty, and Frailty, in a cohort of older Scottish adults.

## 2 METHODS

### 2.1 Study design

This longitudinal prospective study used data from the Lothian Birth Cohort 1936 (LBC1936), which has been described in detail elsewhere.^13^, ^14^ In summary, participants living in the Lothian region of Scotland (which includes Edinburgh), most of whom had completed an intelligence test aged 11 years, were recruited in 2004, at mean age 69.5 years (*N* = 1091). They have been followed up every 3 years since, at mean ages 72.5 years (*n* = 866), 76.3 years (*n* = 697), 79.3 years (*n* = 550) and 82 years (*n* = 431). All participants are white, and the sex split is approximately equal. At each wave, participants undergo interviews, questionnaires, blood tests and physical measures, including a timed gait test over 6 m. LBC1936 is conducted according to all applicable ethical guidelines. Written consent is obtained from participants at each wave.^14^

### 2.2 Participants and study size

**FIGURE 1:**
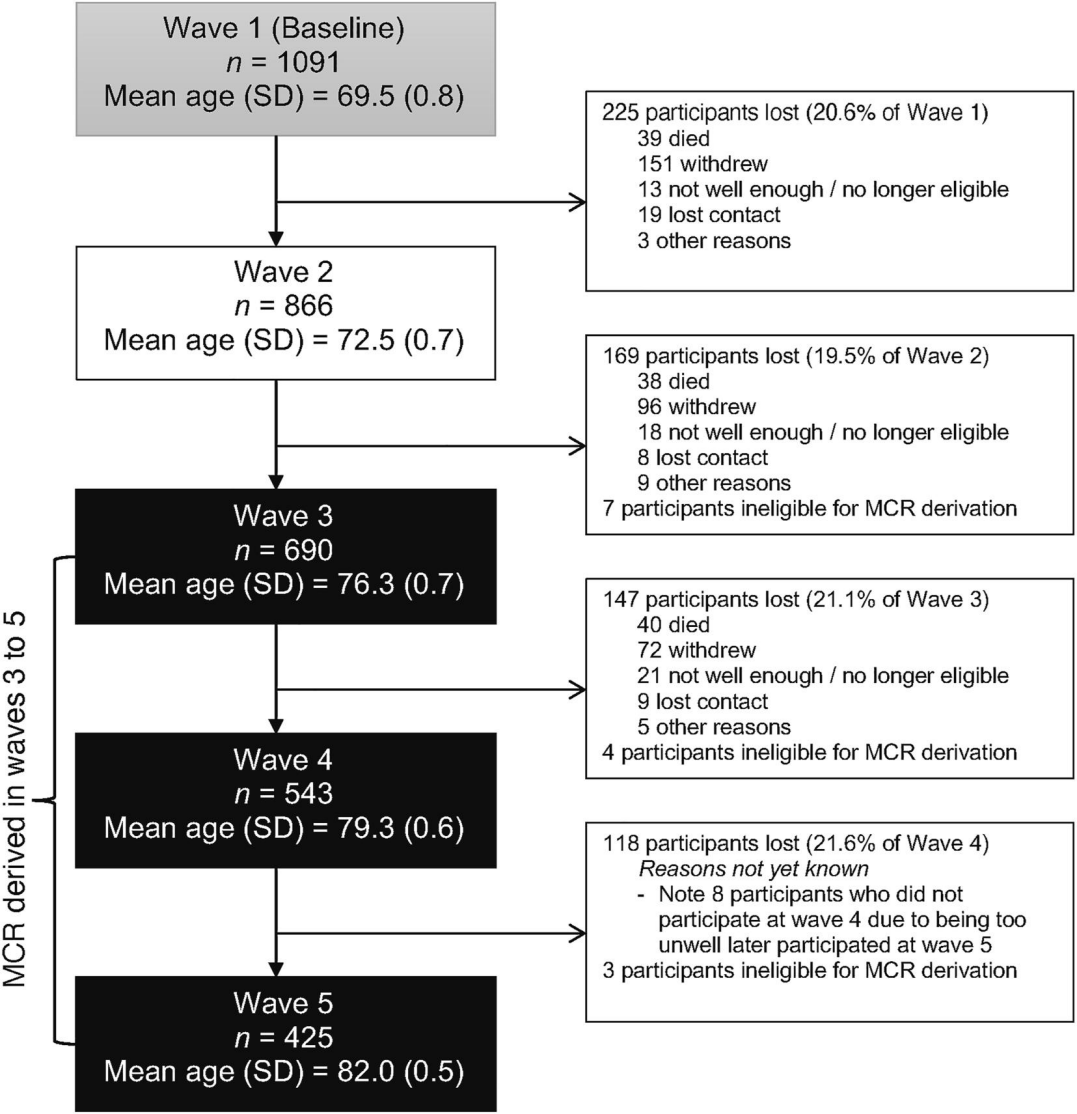
Flow chart of sample selection

For our analysis, we excluded participants with dementia and those missing data required to derive the MCR phenotype. We describe sample selection with reasons for dropout and exclusion given, where known, in Figure 1.

## 3 IDENTIFICATION OF MCR

MCR is defined as the presence of subjective cognitive complaints and objective slow gait in older individuals without dementia or significant functional disability.^3^ To be classified in the MCR category, participants had to meet all four criteria reported below:

1. Slow gait as defined by walking speed greater than or equal to 1 standard deviation (SD) slower than age‐ and sex‐matched means. The time taken (in seconds) to walk 6 m along a corridor was recorded with a stopwatch.^3^
2. Self‐reported cognitive complaint: answering “yes” to the question “do you currently have any problems with your memory?”
3. No diagnosis of dementia: does not self‐report a diagnosis of dementia *and* scores at least 24/30 on the Mini‐Mental State Examination (MMSE).^15^
4. Preservation of independence in functional abilities: less than or equal to 1.5 SD above the mean on the Townsend Disability Scale overall score (a higher score indicates greater disability).^16^

We derived MCR from wave 3 (age 76) onwards, as the variables measuring these criteria were first identified at wave 3.

### 3.1 Covariates

We examined the association between the following baseline (age 69.5 [0.8] years) covariates and MCR status at waves 3, 4, and 5: age, sex, years of education, socioeconomic status, age 11 intelligence quotient (IQ; derived from the Moray House Test), marital status, body mass index (BMI), self‐reported smoking status (current/ex/never), self‐reported alcohol intake (units per week), depression and anxiety symptoms (Hospital Anxiety and Depression Scale), self‐reported history of cardiovascular disease, and stroke. Other physical measures included forced expiratory volume in 1 s (FEV_1_), which is a measure of lung capacity, and grip strength (combined average of left and right). We also compared levels of blood C‐reactive protein (CRP), a non‐specific measure of inflammation, between non‐MCR and MCR groups. All of these covariates were selected a priori as they were previously associated with MCR^8^, ^10^, ^11^ except for age 11 IQ, which was never tested. Higher childhood intelligence has previously been associated with faster gait and less subjective cognitive complaints in mid‐ to later‐life.^17^ See Supplementary Table 1 for more details on how these variables were measured or derived.

### 3.2 MCR overlap with MCI and Frailty levels

MCI (present/absent)^18^ and physical Frailty level according to Fried phenotype (Frail/Prefrail/not frail)^19^ have been derived in the LBC1936, as detailed in Supplementary Table 1. We explored the overlap of these phenotypes with MCR within each wave of the LBC1936 dataset. As slow gait speed is common to MCR and Prefrailty/Frailty, we examined the proportion of those with Prefrailty/Frailty who were slow walkers as per MCR slow gait criterion (≥1 SD below age‐ and sex‐matched mean).

### 3.3 Statistical methods

We used descriptive analyses including the number and percentages of people with MCR to characterise the study sample. We summarised the participants' characteristics using means and SD or frequencies and percentages, as appropriate.

We classified participants into two groups: non‐MCR and MCR. These groups were compared using *χ* ^2^ tests with a continuity correction for categorical variables. For continuous explanatory variables, we performed an *F*‐test (ANOVA) by default. We performed a Kruskal‐Wallis test when variables were considered non‐parametric, except in cases where Fisher's exact test was more appropriate (i.e., when expected counts were less than five).^20^ *p*‐values less than 0.05 were considered statistically significant. Since all covariates were of substantive interest a priori, no adjustment for multiple comparisons was incorporated into the analysis.^21^, ^22^, ^23^ All statistical analyses were conducted in R version 4.0.2.^24^

### 3.4 Subgroup analysis

Common to most prospective longitudinal studies of ageing, LBC1936 is susceptible to sampling bias through attrition.^13^ Compared to individuals who remained in the study, those who dropped out at each wave had lower age‐11 IQ scores, lower Mini‐Mental State Examination (MMSE) scores, lower socioeconomic class, and poorer physical fitness.^13^ To account for this, we performed a subgroup analysis of the MCR prevalence rates and covariates for those who withdrew compared to those who remained in the study.

### 3.5 Missing data

We compared the distribution of all variables with missing data amongst MCR and non‐MCR groups. The LBC1936 researchers try to maintain a low loss to follow‐up rate at each wave by re‐contacting those unable to attend a wave due to a temporary illness and seeing them at a later, more appropriate time where possible.^14^

## 4 RESULTS

Figure 1 illustrates the flow of our sample participants. We excluded three participants who had been diagnosed with dementia by the LBC1936 study doctor before wave 3. The variables necessary for deriving MCR were measured in LBC1936 from wave 3 onwards. Participants missing data in any of the necessary MCR criteria were excluded from analyses at wave 3 (*n* = 4), wave 4 (*n* = 4), and wave 5 (*n* = 3). Accordingly, MCR status was coded for 690 participants at wave 3 (48.0% female, mean age 76.3 years), 543 participants at wave 4 (49.7% female, mean age 79.3 years), and 425 participants at wave 5 (51.1% female, mean age 82 years). Loss to follow‐up in LBC1936 was approximately 20% after each wave. The main reasons for attrition were death, chronic incapacity, and permanent withdrawal.^14^ The participation rate of eligible persons was over 99% at each wave.

### 4.1 MCR prevalence

MCR prevalence was very similar across waves at 5.7% (95% CI 4.0–7.6; *n* = 39/690) at wave 3, 5.3% (95% CI 3.6–7.5; *n* = 29/543) at wave 4%, and 5.4% (95% CI 3.6–8.2; *n* = 23/425) at wave 5. The mean prevalence of MCR was 5.5% (95% CI 4.5–6.7) across three waves with participants aged from 74 to 83 years old. We performed a sensitivity analysis of the MCR prevalence for those who withdrew by wave 5 (‘withdrawers’; *n* = 398) compared to those who remained in the study throughout (*n* = 425). MCR prevalence was higher overall amongst withdrawers at 8.6% (95% CI 5.1–11.6; *n* = 23/269) at wave 3% and 11% (95% CI 5.5–16.1; *n* = 14/127) at wave 4.

The gait speed cut‐offs by age and sex used to define MCR are presented in Table 1. These cut‐offs were established using data from waves 3, 4, and 5, as these were when MCR was derived.

**TABLE 1:** Gait speed cut‐offs by age and sex in this cohort for defining Motoric Cognitive Risk

Age range (mean), years	Wave	Gait speed (m/s)	
		Male	Female
74.6–77.7 (76.2)	3	1.07	1.00
77.7–80.9 (79.3)	4	0.97	0.90
80.9–83.1 (82.0)	5	0.96	0.83

*Note*: Gait speed (m/s) values at or below the cut‐off scores (≥1 standard deviation below age‐ and sex‐matched means) were used to define slow gait. MCR was first derived at wave 3.

### 4.2 Baseline covariate differences

Baseline covariate differences of the participants according to MCR status at wave 3 (6 years follow‐up), wave 4 (9 years follow‐up) and wave 5 (12 years follow‐up) are presented in Table 2. Older age was significantly associated with having MCR at waves 3 and 4, but not wave 5, despite the narrow age range of LBC1936 participants (SD 0.8 years). Sex, years of education, and age 11 IQ were not significantly associated at any wave. Lower socioeconomic status (defined by manual occupation) was significantly associated with MCR at wave 3. At all waves, poorer scores in one or more of the following tests of executive function were significantly associated with MCR compared to non‐MCR: verbal fluency (wave 3), digit‐symbol test (waves 3, 4 and 5), four‐choice reaction time (wave 3), and block design (wave 5). The physical measures of FEV_1_ and average grip strength were significantly associated with MCR outcome at wave 4. BMI, anxiety, and depression symptoms were significantly associated with MCR at wave 5. No further covariate associations were found. There was no significant difference in missing data for any of the variables between MCR and non‐MCR groups.

**TABLE 2:** Baseline covariate differences of the participants according to MCR status at wave 3, wave 4 and wave 5

		MCR at wave 3 (6 years follow‐up)				MCR at wave 4 (9 years follow‐up)				MCR at wave 5 (12 years follow‐up)			
Baseline covariates		No MCR	MCR	Total	*p*	No MCR	MCR	Total	*p*	No MCR	MCR	Total	*p*
Total N (%)		651 (94.35)	39 (5.65)	690		514 (94.66)	29 (5.34)	543		402 (94.59)	23 (5.41)	425	
Age, years	Mean (SD)	69.5 (0.8)	69.9 (0.7)	69.5 (0.8)	**0.0008**	69.5 (0.8)	69.8 (0.7)	69.5 (0.8)	**0.0391**	69.5 (0.8)	69.5 (0.9)	69.5 (0.8)	0.9455
Sex	Male	340 (52.23)	19 (48.72)	359 (52.03)	0.7423	260 (50.58)	13 (44.83)	273 (50.28)	0.5725	200 (49.75)	8 (34.78)	208 (48.94)	0.1999
	Female	311 (47.77)	20 (51.28)	331 (47.97)		254 (49.42)	16 (55.17)	270 (49.72)		202 (50.25)	15 (65.22)	217 (51.06)	
Education, years	Mean (SD)	10.8 (1.1)	10.6 (1.1)	10.8 (1.1)	0.3414	10.9 (1.2)	10.6 (1.2)	10.9 (1.2)	0.2088	10.9 (1.2)	10.5 (1.2)	10.9 (1.2)	0.0837
Age 11 IQ	Mean (SD)	101.6 (15.4)	100.8 (12.2)	101.6 (15.3)	0.7119	101.9 (15.3)	101.7 (15.2)	101.9 (15.2)	0.9497	102.5 (15.0)	103.5 (13.2)	102.5 (14.9)	0.7198
SES	Non‐manual	520 (79.88)	23 (58.97)	543 (78.70)	**0.0056**	417 (81.13)	22 (75.86)	439 (80.85)	0.3269	335 (83.33)	19 (82.61)	354 (83.29)	0.7659
	Manual	123 (18.89)	15 (38.46)	138 (20.00)		88 (17.12)	7 (24.14)	95 (17.50)		60 (14.93)	4 (17.39)	64 (15.06)	
	(Missing)	8 (1.23)	1 (2.56)	9 (1.30)		9 (1.75)	0 (0.00)	9 (1.66)		7 (1.74)	0 (0.00)	7 (1.65)	
Marital status	Married	465 (71.43)	32 (82.05)	497 (72.03)	0.1981	371 (72.18)	17 (58.62)	388 (71.45)	0.1384	288 (71.64)	16 (69.57)	304 (71.53)	0.8149
	Not married	186 (28.57)	7 (17.95)	193 (27.97)		143 (27.82)	12 (41.38)	155 (28.55)		114 (28.36)	7 (30.43)	121 (28.47)	
Smoking status	Never	325 (49.92)	22 (56.41)	347 (50.29)	0.1391	277 (53.89)	17 (58.62)	294 (54.14)	0.4597	209 (51.99)	16 (69.57)	225 (52.94)	0.1784
	Ex‐smoker	283 (43.47)	12 (30.77)	295 (42.75)		216 (42.02)	10 (34.48)	226 (41.62)		179 (44.53)	6 (26.09)	185 (43.53)	
	Current	43 (6.61)	5 (12.82)	48 (6.96)		21 (4.09)	2 (6.90)	23 (4.24)		14 (3.48)	1 (4.35)	15 (3.53)	
Alcohol, units/week	Mean (SD)	10.5 (13.5)	8.8 (9.2)	10.4 (13.3)	0.2708	6.0 (1.0–15.0)	5.0 (0.5–13.0)	6.0 (1.0–15.0)	0.4764	7.0 (1.0–15.0)	3.0 (0.8–10.5)	6.0 (1.0–15.0)	0.1894
CVD history	No CVD	502 (77.11)	27 (69.23)	529 (76.67)	0.2481	394 (76.65)	24 (82.76)	418 (76.98)	0.6498	318 (79.10)	18 (78.26)	336 (79.06)	1.0000
	CVD	149 (22.89)	12 (30.77)	161 (23.33)		120 (23.35)	5 (17.24)	125 (23.02)		84 (20.90)	5 (21.74)	89 (20.94)	
Stroke history	No stroke	628 (96.47)	36 (92.31)	664 (96.23)	0.1767	496 (96.50)	28 (96.55)	524 (96.50)	1.0000	391 (97.26)	22 (95.65)	413 (97.18)	0.4917
	Stroke	23 (3.53)	3 (7.69)	26 (3.77)		18 (3.50)	1 (3.45)	19 (3.50)		11 (2.74)	1 (4.35)	12 (2.82)	
BMI, kg/m^2^	Mean (SD)	27.6 (4.2)	28.2 (4.0)	27.6 (4.2)	0.3697	27.4 (4.2)	28.5 (4.8)	27.5 (4.3)	0.2710	27.3 (4.0)	28.9 (3.7)	27.3 (4.0)	**0.0462**
APOE	No ε4	432 (66.36)	23 (58.97)	455 (65.94)	0.8481	341 (66.34)	19 (65.52)	360 (66.30)	0.8328	276 (68.66)	14 (60.87)	290 (68.24)	0.2336
	ε4 carrier	186 (28.57)	11 (28.21)	197 (28.55)		145 (28.21)	9 (31.03)	154 (28.36)		103 (25.62)	9 (39.13)	112 (26.35)	
	(Missing)	33 (5.07)	5 (12.82)	38 (5.51)		28 (5.45)	1 (3.45)	29 (5.34)		23 (5.72)	0 (0.00)	23 (5.41)	
FEV_1_, L	Mean (SD)	2.5 (0.7)	2.2 (0.7)	2.4 (0.7)	0.0746	2.5 (0.7)	2.1 (0.6)	2.5 (0.7)	**0.0030**	2.5 (0.7)	2.3 (0.6)	2.5 (0.7)	0.1171
CRP, mg/L	Mean (SD)	4.6 (5.6)	6.4 (8.5)	4.7 (5.8)	0.2177	4.6 (5.7)	4.7 (4.4)	4.6 (5.6)	0.8938	4.5 (5.9)	4.0 (4.6)	4.5 (5.9)	0.6606
HADS‐A	Mean (SD)	4.8 (3.1)	5.3 (3.3)	4.8 (3.1)	0.3483	4.8 (3.1)	5.4 (3.2)	4.8 (3.1)	0.3177	4.6 (2.9)	6.3 (3.7)	4.7 (3.0)	**0.0463**
HADS‐D	Mean (SD)	2.7 (2.2)	2.9 (2.2)	2.7 (2.2)	0.4187	2.7 (2.2)	2.8 (1.9)	2.7 (2.2)	0.7159	2.5 (2.0)	3.9 (2.7)	2.5 (2.1)	**0.0187**
Verbal fluency	Mean (SD)	43.5 (12.8)	38.6 (12.7)	43.2 (12.8)	**0.0254**	43.6 (12.7)	41.6 (13.2)	43.5 (12.7)	0.4255	43.7 (12.4)	42.8 (14.9)	43.6 (12.5)	0.7946
Digits backwards	Mean (SD)	8.0 (2.4)	7.6 (2.2)	7.9 (2.3)	0.3271	8.1 (2.4)	7.6 (2.0)	8.0 (2.3)	0.2261	8.2 (2.4)	7.5 (1.7)	8.1 (2.3)	0.0728
Block design	Mean (SD)	35.2 (10.1)	33.0 (9.9)	35.1 (10.1)	0.1784	35.8 (10.2)	32.3 (9.2)	35.6 (10.2)	0.0605	36.4 (9.9)	31.4 (10.1)	36.1 (9.9)	**0.0307**
Digit symbol	Mean (SD)	58.8 (12.4)	51.2 (11.5)	58.3 (12.5)	**0.0002**	59.3 (12.5)	52.9 (10.8)	58.9 (12.4)	**0.0041**	60.3 (12.2)	56.5 (8.1)	60.1 (12.1)	**0.0448**
Simple reaction	Mean (SD)	0.3 (0.1)	0.3 (0.1)	0.3 (0.1)	0.0859	0.3 (0.1)	0.3 (0.0)	0.3 (0.1)	0.2529	0.3 (0.0)	0.3 (0.0)	0.3 (0.0)	0.9971
4‐choice reaction	Mean (SD)	0.6 (0.1)	0.7 (0.1)	0.6 (0.1)	**0.0101**	0.6 (0.1)	0.6 (0.1)	0.6 (0.1)	0.2773	0.6 (0.1)	0.6 (0.1)	0.6 (0.1)	0.2180
Grip strength, kg	Mean (SD)	29.0 (9.7)	26.8 (10.3)	28.9 (9.7)	0.2043	28.9 (10.0)	24.5 (7.8)	28.7 (9.9)	**0.0069**	28.9 (9.6)	25.9 (10.3)	28.7 (9.6)	0.1940

*Note*: Bold values indicates those with *p*‐values < 0.05, i.e. statistically significant.

Abbreviations: ApoE4, Apolipoprotein ε4 allele; BMI, Basal Metabolic Rate; CRP, C‐reactive Protein; CVD, Cardiovascular; FEV_1_, Forced Expiratory Volume in 1 s; HADS‐A, Hospital Anxiety and Depression Status—Anxiety; HADS‐A, Hospital Anxiety and Depression Status—Depression; IQ, Intelligence Quotient; kg/m^2^, kilogrammes per metre squared; L, litre; MCR, Motoric Cognitive Risk; mg/L, milligrammes per litre; *p*, *p*‐value (0.05 significance); SD, Standard Deviation; SES, Socioeconomic Status.

### 4.3 Subgroup analysis

We performed a sensitivity analysis of the same baseline covariate differences according to MCR status of withdrawers before wave 4 (Supplementary Table 2) and wave 5 (Supplementary Table 3) to assess for selection bias due to attrition. We used an identical statistical approach as for the main analysis. Only verbal fluency at wave 3 (*p* = 0.015) and FEV1 (*p* = 0.0079) at wave 4 differed significantly between the non‐MCR and MCR groups.

### 4.4 MCR, MCI and Frailty level overlap

**FIGURE 2:**
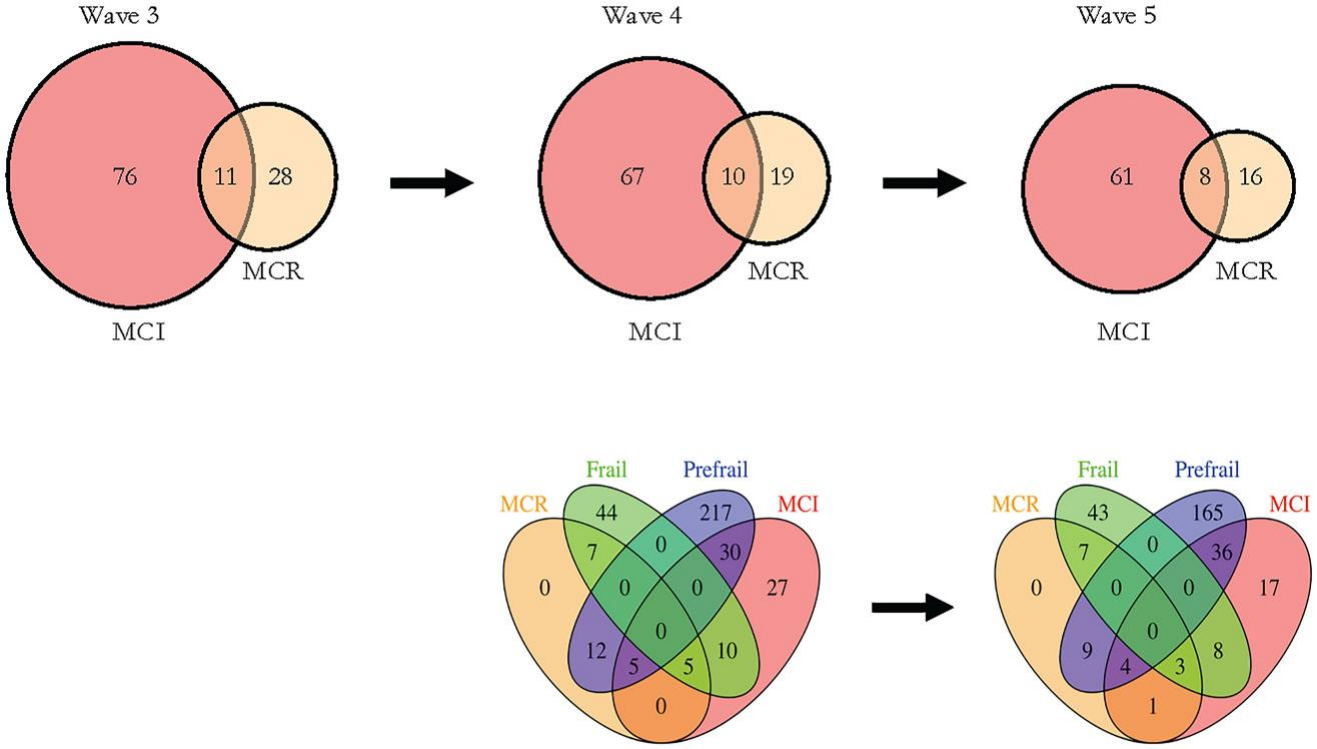
The overlap between Motoric Cognitive Risk (MCR), Mild Cognitive Impairment (MCI) and Frailty level in the LBC1936 cohort. Top, MCR and MCI overlap at waves 3, 4 and 5. Bottom, MCR, MCI, Frailty and Prefrailty overlap at waves 4 and 5. Frailty and Prefrailty were not measured at wave 3 due to lack of necessary variables. By definition, participants cannot be classed as Frail and Prefrail simultaneously

The overlap between MCR, MCI, Prefrailty, and Frailty is presented in Figure 2. MCI was derived at waves 3, 4 and 5 but Frailty level was only derived at waves 4 and 5 due to the unavailability of necessary variables at wave 3.^19^ As a proportion of those participants with either MCR or MCI, the overlap between MCR and MCI is remarkably consistent across each wave—10.6% at wave 3, 11.6% at wave 4%, and 10.4% at wave 5, averaging 10.9% (95% CI 7.4–15.2) across all three waves. Of those participants with MCR, overlap with MCI is 39.3% at wave 3, 52.6% at wave 4%, and 50% at wave 5, averaging 46% (95% CI 33.4–59.1) across all three waves. Only one participant with MCR does not have either Prefrailty or Frailty at waves 4 and 5. The proportion of individuals with Prefrailty or Frailty who met the MCR definition of slow walking was 15.9% (42/264; Prefrail at wave 4), 12.6% (27/214; Prefrail at wave 5), 57.6% (38/66; Frail at wave 4), and 54.1% (33/61; Frail at wave 5).

## 5 DISCUSSION

### 5.1 MCR prevalence

In this cohort of older Scottish adults, we have determined the prevalence of MCR syndrome, described associated risk factors and assessed its overlap with Mild Cognitive Impairment, Prefrailty, and Frailty. The prevalence of MCR averaged 5.5% (95% CI 4.5–6.7) over three waves with participants aged from 74 to 83 years old. There was no significant difference between men and women. This prevalence is lower than the 9.7% pooled rate of 22 cohorts from 17 countries, mean age 73.6 years (±8.2).^5^ This may be partly explained by attrition bias, as the MCR prevalence of withdrawers at waves 3 and 4 is higher than completers. Interestingly though, two studies of our closest neighbours, The English Longitudinal Study of Ageing^5^ and The Irish Longitudinal Study on Ageing^25^ reported rates of 2% and 2.6% respectively. LBC1936 MCR prevalence may be higher than those of the English and Irish cohorts due to the older average age of the LBC1936 participants and the accompanying increased rates of cognitive complaints.

The gait speed cut‐offs in our study were higher in men than women, and lower with older age, in keeping with the literature.^26^ Our gait speed cut‐offs were higher than most reported in other studies of MCR for each age‐ and sex‐matched group.^3^, ^6^, ^27^ In fact, the slow gait cut‐offs in LBC1936 were similar to the mean usual gait speeds for similar groupings in a comprehensive meta‐analysis of usual gait speeds of 23,111 individuals from 12 countries.^26^ This could indicate that the average usual walking speed is quite fast in Scotland but more likely reflects the level of health in LBC1936 participants. Without published national reference age‐ and sex‐matched gait speeds, it is difficult to be sure.

### 5.2 Baseline covariate differences

Interestingly, despite the narrow age range of LBC1936 participants, we noted a significant association between older age and the presence of MCR in two of the three waves. This mixed picture is in keeping with a recent meta‐analysis of factors associated with MCR, which found that the majority but not all of the 22 studies reported age as an associated factor for the presence of MCR.^10^

Lower socioeconomic status, as defined by having had a manual occupation, was associated with having MCR later in life. Individuals who are more physically active while working in a manual job should experience less gait speed slowing in later life and thus a reduced likelihood of having MCR.^28^ However, it is possible that the less cognitively demanding nature of manual work overrides any protective effects of being more physically active at work,^29^ resulting in a net increase in MCR prevalence in individuals with lower socioeconomic status.

It is perhaps unsurprising that MCR was consistently associated with poorer scores in tests of executive function across the three waves as slow gait speed has been repeatedly associated with these tests in the literature.^30^ One hypothesis is that walking requires significant top‐down coordination and planning as well as attention and response inhibition, particularly when walking in an unfamiliar environment.^30^ Indeed, imaging studies have shown that the brain areas most responsible for executive function tasks are often more damaged in the MCR group than in the non‐MCR group.^7^, ^31^, ^32^ In particular, the digit symbol test was the only covariate to remain significant across all three waves (wave 3 *p* = 0.0002, wave 4 *p* = 0.0041, wave 5 *p* = 0.0448), highlighting it as an especially sensitive marker of MCR. The digit symbol test, a subtest from the Wechsler Adult Intelligence Scale‐III UK,^33^ predominantly assesses processing speed. An age‐related reduction in processing speed has long been recognised as the most commonly affected cognitive ability with ageing^34^ and a leading indicator of changes in memory in older adults.^34^, ^35^ Decreased processing speed may increase the likelihood of experiencing subjective cognitive problems, and thus MCR diagnosis. In the digit symbol test, the participant enters a symbol according to a given number‐symbol code, completing as many as possible in 2 min. A higher score indicates a better performance. This test has been previously found to serve as a biomarker of risk of clinical disorders of cognition and mobility.^36^

This is the first study to examine the association between early‐life intelligence test score and MCR status later in life. There was no significant relationship found. This is an important finding as it does not support previous work detailing an association between lower early life intelligence scores and slower gait and poorer cognitive performance.^17^ Consistent with the literature, alcohol consumption was also not significantly associated with MCR status.^8^, ^10^, ^11^ More surprisingly, however, were the findings that years of education, stroke, and cardiovascular disease were not associated with MCR status. These covariates have generally been associated with MCR, even in cohorts with similarly high education levels as LBC1936.^8^, ^10^, ^11^, ^25^ The lack of association between MCR and BMI, depression, and anxiety at waves 3 and 4, or grip strength at waves 3 and 5 could be due to a combination of small effect sizes and our relatively small study size. This idea is supported by our effect sizes which, although not significant, are generally in the same direction as larger studies.^8^, ^10^, ^11^ Additionally, this variation may be because the LBC1936 cohort consists of participants from an affluent area of Scotland who volunteered to take part, and the average years of education, as well as general physical fitness, is notably higher than the general population.^13^, ^14^ This healthy volunteer bias is common to longitudinal studies of ageing and should be considered when deciding how generalisable our findings are to the clinical population.

### 5.3 Overlap of MCR, MCI, Prefrailty and Frailty

The limited degree of overlap between MCR and MCI in our study shows that these two concepts, although derived using similar criteria and thus sharing some participants, also capture different cohorts of people. Many participants with MCI do not have MCR. This may be partly because gait speed slows as early as 10 years before the diagnosis of MCI and some literature suggests that slow gait may precede declines in cognitive function tests.^37^ A recent study comparing MCR and MCI found many shared risk factors but also noted that differences in gender, hypercholesterolaemia, BMI, and cerebral white matter volumes indicate different pathophysiological substrates.^38^ This study also reported that MCR captured early features of dementia in the absence of MCI.^38^

This is the first study to explore the overlap of MCR and Frailty according to Fried phenotype (characterised as the presence of five components: weakness, slowness, exhaustion, low physical activity, and unintentional weight loss).^39^ Given the degree of overlap found, it is surprising that individual characteristics of Frailty, such as grip strength and BMI were not significantly different between the MCR and non‐MCR groups at all waves. The overlap must be largely explained by the other frailty criteria. Over half of the individuals classed as Frail were slow walkers by MCR standards. We used the standard MCR approach to define slow gait speed as ≥1SD (i.e., 15.1%) slower than the age‐ and sex‐matched mean. When calculating the Fried Frailty phenotype in LBC1936, slow gait was defined using a method typical of frailty papers—“the slowest sex‐ and height‐adjusted 20% of the distribution.”^19^ This distinction may explain some of the differences between the MCR group and slow‐walking Frail/Prefrail individuals. The more stringent MCR slow gait definition partly explains the relatively high slow gait cut‐offs despite a high prevalence of Frailty and Prefrailty in the sample.

### 5.4 Implications

Our findings have several implications. First, if the association between MCR and lower socioeconomic status reflects a causal link, policy measures which target socioeconomic inequality during people's working life might reduce the numbers of individuals transitioning to MCR and then to dementia. Second, our findings regarding the overlap between MCR and MCI reinforce previous research which determined that we should view MCR as complementary to MCI rather than replacing it.^3^ Assessing for both prodromes is likely to yield more people at high risk of developing dementia.^3^ Third, the decision as to whether MCR, MCI, Prefrailty, or Frailty is more clinically useful will ultimately be determined by balancing the cost, effort and time taken to measure each prodrome with the prognostic value for the outcome in question.

Our data had limitations in addition to those already discussed. On the one hand, our sensitivity analysis comparing the baseline covariates differences according to the MCR status of withdrawers before wave 4 and wave 5 was reassuring as there were very few significant differences. However, the withdrawal rate for individuals with MCR was significantly higher than for those without MCR, indicating likely selection bias due to attrition. Attrition from ill‐health or mortality is a common and often unavoidable bias of longitudinal studies of ageing. Despite attempts to minimise attrition by rescheduling wave appointments for individuals unable to attend due to illness, the attrition rate between waves in the LBC1936 is approximately 20%. This is at the upper limit of what is considered acceptable by international quality assessment groups.^40^ Finally, our small sample size means a replication study in a larger cohort, or a cohort with a higher prevalence of MCR, would increase confidence in our findings.

## 6 CONCLUSION

Prevalence rates of MCR in this Scottish cohort are lower than the global average but higher than in neighbouring countries. Future Lothian Birth Cohort 1936 research should assess the risk factors associated with MCR to validate previous findings and analyse novel predictive factors. Work exploring the association between socioeconomic status and MCR could help address the disparities in health care and health outcomes in the United Kingdom. Examining the prognostic value of MCR as a predictor of cognitive decline, specifically executive function, in LBC1936 is a vital avenue to explore. Our study can serve as a foundation for future studies to improve dementia risk assessments and potentially develop new interventions to reduce incident dementia.

## AUTHOR CONTRIBUTIONS

Donncha S. Mullin, Graciela Muniz‐Terrera, Tom C. Russ, and Michelle Luciano generated the idea for the present manuscript. Donncha S. Mullin obtained and analysed the data, draughted the manuscript, and is the guarantor. Miles Welstead and Lucy E Stirland provided additional data and code. All authors edited the manuscript and gave final approval of the version to be published. The corresponding author attests that all listed authors meet authorship criteria and that there has been no omission of others meeting the criteria.

## CONFLICTS OF INTEREST

The author declares that there is no conflict of interest that could be perceived as prejudicing the impartiality of the research reported.

## Supporting information

Supporting Information S1

## Data Availability

The data that support the findings of this study are available from the corresponding author upon reasonable request.
